# Draft Genome Sequences of Five Strains of *Lactobacillus acidophilus*, Strain CIP 76.13^T^, Isolated from Humans, Strains CIRM-BIA 442 and CIRM-BIA 445, Isolated from Dairy Products, and Strains DSM 20242 and DSM 9126 of Unknown Origin

**DOI:** 10.1128/genomeA.00658-13

**Published:** 2013-08-22

**Authors:** Hélène Falentin, Sylvie Cousin, Dominique Clermont, Sophie Creno, Laurence Ma, Victoria Chuat, Valentin Loux, Pukall Rüdiger, Chantal Bizet, Christiane Bouchier

**Affiliations:** INRA, UMR1253 Science et Technologie du Lait et de l’OEuf, Rennes, Francea; Agrocampus Ouest, UMR1253 Science et Technologie du Lait et de l’OEuf, Rennes, Franceb; Institut Pasteur, Centre de Ressources Biologiques de l’Institut Pasteur, Département de Microbiologie, Paris, Francec; Institut Pasteur, Plate-forme Génomique, Département Génomes et Génétique, Paris, Franced; INRA, UR1077 Mathématique, Informatique et Génome, Jouy-en-Josas, Francee; Leibniz-Institut DSMZ - Deutsche Sammlung von Mikroorganismen und Zellkulturen GmbH, Braunschweig, Germanyf

## Abstract

*Lactobacillus acidophilus* is a natural inhabitant of mammalian gastrointestinal systems and is used in dairy and pharmaceutical products. Five draft genome sequences, covering 1,995,790 nucleotides (nt) on average, are divided into 19 to 34 scaffolds covering 1,995 to 2,053 genes. The draft genome sequences were compared to the sequence of the *L. acidophilus* NCFM dairy strain.

## GENOME ANNOUNCEMENT

*Lactobacillus acidophilus* is an important natural inhabitant of mammalian gastrointestinal systems (1). *L. acidophilus* has been described as playing a specific role in human health and nutrition by stabilizing the normal intestinal microflora. Consequently, it is used in the industrial preparation of dairy and pharmaceutical products as a source of dietary lactobacilli.

Here, we report the draft genome sequences of 5 strains of *L. acidophilus,* CIP 76.13^T^, DSM 9126, DSM 20242, CIRM-BIA 442, and CIRM-BIA 445, obtained using a whole-genome strategy based on Illumina paired-end sequencing with an average insert length of about 458 bp (Illumina genome analyzer HiSeq 2000) observed on the Agilent high-sensitivity DNA kit. The numbers of quality-filtered reads cover 11.1, 8.4, 9.5, 15.1, and 10.3 Gb per genome, respectively (88.6 bases mean read length, ~464-fold average coverage). The reads were assembled using ABySS version 1.2.6 (2) or Velvet version 2.2.0 (3) softwares. The N_50_ varied from 166,831 to 242,633 bases.

The draft genome sequences of CIP 76.13^T^, DSM 9126, DSM 20242, CIRM-BIA 442, and CIRM-BIA 445 consist of 1,995,790 nucleotides (nt) on average (varying from 1,951,821 to 2,047,898), divided into 34, 26, 20, 19, and 22 scaffolds, respectively. All strains had a G+C content of 36%. The genome sequences were annotated with the AGMIAL platform (4). For *L. acidophilus* CIP 76.13^T^, DSM 9126, DSM 20242, CIRM-BIA 442, and CIRM-BIA 445, the predicted (i) gene number, (ii) number of rRNA gene copies (in parentheses: 5S, 23S, and 16S), and (iii) number of tRNA genes were 2,036 (1 each) and 58, 2,030 (1, 1, 0) and 52, 2,053 (3, 3, 5) and 57, 1,995 (2, 2, 4) and 58, and 2,049 (1 each) and 44, respectively. The sequence of each genome has been compared to that of the NCFM strain genome (5) with PROmer from MUMmer package (6) and KoriBlast (Korilog SARL) at the protein level.

Compared to NCFM strain (5), DSM 9126 and CIRM-BIA 442 lack the potential autonomous unit (pauLA-III), whereas CIRM-BIA 445 lacks a locus containing several glucosidases and a cellobiose phosphotransferase system (PTS). The genomes of CIRM-BIA 445, DSM 9126, and DSM 20242 contain a locus encoding peptidase, aminocyclase, aspartate transporter, exopolyphosphatase, and polyphosphate kinase that is not present in the NCFM genome. In all studied strains, several genes appeared pseudogenized, notably *comFC* (involved in competence) and *glgC* (involved in glycogen metabolism) in CIRM-BIA 445, *epsF* (involved in exopolysaccharide synthesis) in DSM 9126, *fruA* (involved in fructose transport) in CIRM-BIA 445, the gene encoding aspartate ammonia ligase in DSM 9126, the gene responsible for cell wall teichoic acid glycosylation in CIP 76.13^T^, the gene encoding aspartate semialdehyde dehydrogenase in CIP 76.13^T^, and *rbsK* (involved in ribose catabolism) in CIP 76.13^T^.

### Nucleotide sequence accession numbers. 

The type strain is publicly available in two European Biological Resource Centers under the number CIP 76.13^T^ (and DSM 20079^T^), CIRM-BIA 442 and CIRM-BIA 445 are available at the International Centre of Microbial Resources for Bacteria of Food Interest, and DSM 20242 and DSM 9126 are available at the DSMZ-German Collection of Microorganisms and Cell Cultures. The draft of the whole-sequencing genome project has been deposited in EMBL under the accession no. CBLQ010000001 to CBLQ010000055, CBLP010000001 to CBLP010000019, CBLR010000001 to CBLR010000041, CBLT010000001 to CBLT010000021, and CBLS010000001 to CBLS010000045, for CIP 76.13^T^, CIRM-BIA 442, CIRM-BIA 445, DSM 20242, and DSM 9126, respectively. The versions described in this paper are the first versions.
